# A simple transfer function for nonlinear dendritic integration

**DOI:** 10.3389/fncom.2015.00098

**Published:** 2015-08-10

**Authors:** Matthew F. Singh, David H. Zald

**Affiliations:** ^1^Department of Psychology, Vanderbilt UniversityNashville, TN, USA; ^2^Department of Psychiatry, Vanderbilt UniversityNashville, TN, USA; ^3^The Program in Neurosciences, Washington University School of Medicine in St. LouisSt. Louis, MO, USA

**Keywords:** dendrite, transfer function, neural network, NMDA spike, pyramidal cell

## Abstract

Relatively recent advances in patch clamp recordings and iontophoresis have enabled unprecedented study of neuronal post-synaptic integration (“dendritic integration”). Findings support a separate layer of integration in the dendritic branches before potentials reach the cell's soma. While integration between branches obeys previous linear assumptions, proximal inputs within a branch produce threshold nonlinearity, which some authors have likened to the sigmoid function. Here we show the implausibility of a sigmoidal relation and present a more realistic transfer function in both an elegant artificial form and a biophysically derived form that further considers input locations along the dendritic arbor. As the distance between input locations determines their ability to produce nonlinear interactions, models incorporating dendritic topology are essential to understanding the computational power afforded by these early stages of integration. We use the biophysical transfer function to emulate empirical data using biophysical parameters and describe the conditions under which the artificial and biophysically derived forms are equivalent.

## Introduction

**Figure 1:**
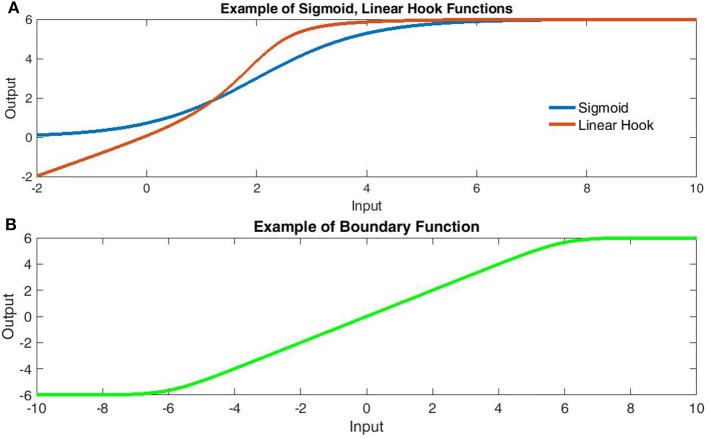
**Comparison of transfer function components**. **(A)** Comparison of sigmoidal (Blue) and Linear Hook functions (Red). The linear hook is a bounded sum of linear and sigmoidal functions [Bound(x + σ(x))]. Note that the sigmoid function fails to capture the subthreshold linearity. **(B)** The linear-boundary function with limits at ±6. The linear boundary function applies soft edges to a linear component and is formed by taking the area under the difference of sigmoids.

Over the past decade, increasing evidence indicates that dendritic architecture plays an active role in shaping somatic responses to synaptic input. Particularly in pyramidal neurons (e.g., Schiller et al., 2000; van Elburg and van Ooyen, 2010; Branco and Häusser, 2011), conceptualizations of the dendritic arbors have shifted from organizational topologies to primary units of computation with unique integration properties that challenge most network abstractions of biological neurons (Häusser and Mel, 2003; Spruston and Kath, 2004; Branco and Häusser, 2010, 2011). From the beginning of computational modeling, network neurons (or “nodes”) have been described as non-linear integrators (often sigmoidal) of linear input. Most commonly, this translates into a nonlinear transform of the global sum of synaptically weighted input (inner-product of an input and weight vector). However, an increasing body of evidence suggests non-linear summation between relatively close inputs within a dendritic branch. For pyramidal neurons, these appear linear for weak inputs, highly super-linear for intermediate inputs and slightly sub-linear for strong inputs (Polsky et al., 2004; Branco and Häusser, 2011). Suggested bio-mechanisms focus upon regenerative branch spikes involving Na^+^, Ca^+^, and/or NMDA spikes (Schiller et al., 2000; Polsky et al., 2004; Antic et al., 2010). Fortunately, this effect becomes increasingly linear as the distance between inputs increases and when summation occurs between branches, suggesting a first layer of non-linear within-branch integration followed by a global integrator of their summed output (Polsky et al., 2004; Spruston and Kath, 2004). This framework has sometimes likened a single neuron to a two or three-layer neural network with the outer (dendritic) layers all converging upon a single (somatic) node (Häusser and Mel, 2003; Polsky et al., 2004; Spruston and Kath, 2004). However, within this metaphor, the literature has consistently referred to sigmoidal dendritic integrators that do not fully match data. In fact, a sigmoidal function quickly generates implausible scenarios such as extremely limited ranges of inhibitory post-synaptic potentials (IPSP's). This is due to the fact that the sigmoid is anti-symmetric about its mid-point near a peak excitatory post-synaptic potential (EPSP) amplitude of 4 mV measured at the soma (Polsky et al., 2004). Moreover, the sigmoid's anti-symmetry implies both sides of the midpoint must be equally linear, which does not allow the observed sub-threshold linearity with extreme nonlinearity post-threshold (see Figure 1A). Rather, the data most resemble a monotonic “hook,” which some have more accurately described as linear-nonlinear with the nonlinear segment concave (Jadi et al., 2014). Based upon current data of subthreshold linearity, it appears the sigmoid's resemblance is only due to oversampling the function's nonlinear upper half (specifically only positive inputs).

In contrast to the oft described “sigmoid,” Poirazi et al. (2003) produced a two-layer model with a binomial-logistic hybrid function of synaptic activation count that resembles a “linear hook” within certain boundaries. This study provided some of the first evidence that a two-layer network (with a non-sigmoidal input layer) can approximate the firing frequency of a detailed model pyramidal cell (see **Figure 6B**). Importantly, Poirazi et al.'s (2003) model used the same linear-hook type function for each dendrite-to-soma transfer prior to a global sigmoidal transform. Results firmly established that simple linear-concave functions of binary input form an adequate input layer to describe firing rates (after a sigmoidal global transform). However, many applications involve continuous metrics of synaptic input or dynamic somatic compartments as in bursting behavior. These situations require information about membrane potentials rather than converting the number of glutamatergic synapses activated into firing rates. Here we use the separation principle of fast-slow dynamics (Genet and Delord, 2002; Wainrib et al., 2012) to derive simple, artificial and biophysical dendritic transfer functions for changes in somatic membrane potential. The biophysical transfer function is then compared to experimental data. Both versions of the transfer function are linear-sigmoid hybrids and hence computationally simple. This is notable because most current models use a single dendritic compartment (or none) for computational simplicity as individual branch models drastically increase processing time. However, the use of time-independent transfer functions removes this barrier as a single nodal compartment may then integrate the non-linear dendritic components. Rather than simulating each branch with a dynamical system of membrane potential, a suitable transfer function may directly convert dendritic input to the induced somatic potential.

## Methods

### General transfer function

We begin by characterizing the dendritic transfer function T_D_(V):

1. As the distance between input sites increases, *T* should become the linear sum.
2. At close distances, *T* is linear for weak inputs, super-linear for intermediate inputs, and slightly sub-linear for strong inputs.
3. Three currents must be accounted for: fast ionic currents (I_fast_), leak current (I_leak_), and a slow NMDAR-mediated current (I_NMDA_).

Biophysical models have made use of the fast-slow dynamics of dendritic membrane to neglect relaxation times of fast channels, instead keeping them constant at equilibrium conductance (Genet and Delord, 2002). To remain time-independent (necessary for a transfer function), we model the net hyper/depolarization for a set of proximal inputs [T_D_(x_1_, x_2_, x_3_,…)] using the distances between input sites as a proxy for time in determining an expectation for leak and NMDAR-mediated currents. The change in potential (relative to base) is then expressed as a bounded sum of linear inputs and nonlinear NMDAR-mediated currents.

As previously mentioned, the sigmoid function does not converge to the linear summation observed for inter-branch dendritic currents. Instead we make use of a juxtaposition of sigmoid integrals of the total polarization to form a locally linear function *G(V)* with upper and lower bounds *b*_*u*_*, b*_*l*_.

(1) $$\begin{array}{l} 0<\alpha_{L,U},\;b_{L}<b_{U,}G\left(\text{V}\right)\\ \;=\int \frac{1}{1+e^{-\alpha_{L}\left(V\;-b_{L}\right)}}-\frac{1}{1+e^{-\alpha_{U}\left(\;V-b_{U}\right)}}dV\\ \;G(V)=ln\left(\frac{{\left(1+e^{\alpha_{L}\left(V\;-b_{L}\right)}\right)}^{\frac{1}{\alpha_{L}}}}{{\left(1+e^{\alpha_{U}\left(V\;-b_{U}\right)}\right)}^{\frac{1}{\alpha_{U}}}}\right)+b_{L} \end{array}$$

Here α_*L*_ and α_*U*_ are the curvature of lower and upper boundaries respectively, while *b*_*L*_ and *b*_*U*_ are the lower and upper boundaries with the constant *b*_*L*_ added to center the function (Figure 1B). Throughout, all potentials are translated so that *E*_Leak_ = 0 for the leak potential. Using the multivariate logistic-sigmoid: σ(X_*D*_):R^*n*^ → R = [1 + exp(−∑{X_i_})]^−1^ for input vector *X*_*D*_ we first describe a simple transfer function which, as can be seen in Figure 1A, qualitatively provides a superior fit relative to a simple sigmoid:

(2) $$T_{Artificial}\left(X\right)=G\left(c_{d}\sigma \left(a_{d}\left[{\hat{X}}_{D}-b_{d}\right]\right)+\sum X_{i}\right)$$

This naïve form simply takes the boundary of the sum of linear and sigmoidally-nonlinear components from the dendritic input vector *X* with *c*_*d*_ the nonlinear maximum, *a*_*d*_ the curvature, and *b*_*d*_ the mid-point (related to threshold) of the nonlinear component (Figure 1A). Although not biophysical, this form bears substantial resemblance to empirical dendritic integration (e.g., Polsky et al., 2004) in its linear-hook appearance and is at least an improvement on linear-integration when few parameters are known. It must be noted that, the artificial transfer function does not consider the locations of input. However, we will now derive a biophysically-reasonable description of dendritic integration for which the naïve form becomes a specific case.

### Biophysical transfer function

**Figure 2:**
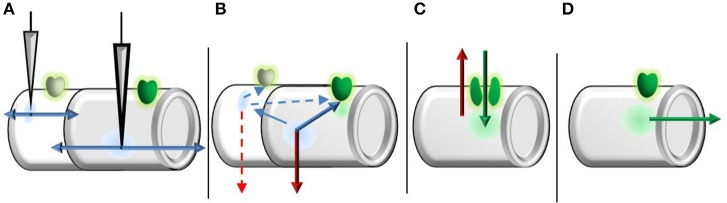
**Graphical representation of time-course separation used to derive the transfer function**. **(A)** Fast ionic (linear) components exit the compartment near instantly and propagate bidirectionally. **(B)** Prior to NMDAR opening, local potentials (within a compartment) are subject to temporal decay (leak current). Starting potentials at channel opening are then formed by spatially decayed fast ionic currents from other compartments and temporally decayed potentials from the relevant compartment. The length of duration prior to opening is on the order of single channel kinetics, as a single channel is sufficient to trigger bursting/clustering behavior. **(C)** While the NMDAR system is active, only two currents are considered: leak current (red) and NMDAR current (green). The length of NMDAR system activation is on the much larger order of burst/cluster length to represent macroscopic currents, rather than the far shorter individual channel open durations. **(D)** After the NMDAR system inactivates, NMDAR currents propagate toward the soma under spatial decay.

To approximate peak EPSP amplitude as a function of input only, we make use of hierarchical dendritic time scales with the separation principle. In this approach, systems with slow and fast components are separated into a fast subsystem, in which the slow variables are held constant, and a slow subsystem contained in the fast nullcline. In the current case, the “linear” fast ionic currents stem from channels with substantially shorter opening times than NMDAR's, while the opening of NMDAR's and Mg^2+^ unblocking is many orders quicker than channel closing (Jahr and Stevens, 1990a,b). As such, we consider the peak EPSP a sum of passively propagating fast ionic currents and dynamically generated NMDA spikes, mirroring the experimental separation of “linear” (fast ionic) and “nonlinear” (spike) components (Figure 2). To remain time-independent, inputs are viewed in terms of the induced local depolarization (*v*_*i*_) as in neurotransmission and brief current pulses. Throughout, vectors are ordered from the least to most distal dendritic segments and all potentials are translated for a resting potential of zero.

#### Spatial decay

Because the fast ionic current is propagated passively, we consider it subject to spatial decay only. Decay is characterized by the functional length constant (λ), which is an empirical parameter derived by fitting attenuation data to a negative exponential of distance. Hence the attenuation from spatial decay, denoted φ(*x_*j*_→_*i*_*) is a negative exponential of the intervening distance:

(3) $$\varphi \left(x_{j\to i}\right)=e^{-\frac{\left|x_{j}-x_{i}\right|}{\lambda }}$$

The functional length constant should not be confused with Rall's (1969) length constant for an infinite cable at steady state. In all simulations we used a length constant for arrival in spike generation half that of the length constant for reaching the soma. This method ensures that stimulus separation has more influence over dendritic spike threshold than passive somatic transients. Similarly, experimental findings demonstrate that increasing inter-electrode distance by a few tens of microns has enormous influence on the spike generation threshold (e.g., Polsky et al., 2004), while the changes in attenuation over that distance should be marginal.

#### Local potential

For simplicity, we divide the NMDAR system into binary open and closed phases and take expectations based on open/close time distributions to transition between phases (Figure 2). While the duration prior to initial opening is considered based on single channel kinetics, the system's open period is defined by the resulting macroscopic current. This dissociation is based upon findings that single NMDAR activations may elicit cluster activation (Gibb and Colquhoun, 1991). Because NMDAR-mediated bursts and clustering determine the resulting macroscopic current dynamics, rather than isolated channels, we consider the NMDAR system's activation duration on the order of burst/cluster lengths as opposed to the brief openings of individual channels (Wyllie et al., 1998). Thus, the duration prior to NMDAR system activation is based on short single channel kinetics, while the period of activation is based on long macroscopic dynamics. Prior to NMDAR opening we consider two sources of depolarization: local and nonlocal input, both of which are subject to decay. Local inputs are subject only to temporal (leak) decay, while we only consider spatial decay for nonlocal inputs prior to channel opening. Letting the closed times (prior to opening) be multi-exponentially distributed (Gibb and Colquhoun, 1991, 1992; Wyllie et al., 1998), the expected leak constant at channel opening is then:

(4) $$\varphi_{i}:\sum_{i=1}^{k}\theta_{i}\frac{R_{m}C}{\tau_{i}+R_{m}C}$$

Here *C* denotes membrane capacitance, τ_*i*_ are the time constants of each exponential, θ_*i*_ the associated amplitudes, and the product *R*_*m*_*C* gives the time constant of temporal decay. Repolarization due to leak current is represented in its usual linear differential equation, producing a solution proportional to the initial condition (as with spatial decay). Because Equations (3) and (4) represent constants of spatial and temporal decay each case requires only a single computation. Ordering segments from least to most distal, potentials at channel opening: *v(t*_*ON*_*)*, may then be represented with a computationally efficient linear matrix equation of the input vector *v(0)*:

(5.1) $$v\left(t_{ON}\right)\sim \Phi v(0)$$

(5.2) $$\Phi_{i,\;j}:=\{\begin{array}{cc} \varphi (x_{j\to i}) & i\neq j\\ \varphi_{i} & i=j \end{array}\;$$

This matrix is symmetric with diagonals corresponding to the constants of temporal decay while nondiagonals correspond to the constants of spatial decay when currents propagate from more distal locations(*i*<*j*) and backpropagate (*i*>*j*). As backpropagation is generally considered more efficient we consider the possibility of differential length constants in Section Varying Distance, although both cases are exponential form of Equation (3). In computing NMDA spikes we use the following alternative notation for brevity in which temporal dynamics start at channel opening:

(6) $$V\left(x_{i},0\right):=V_{0}\left(x_{i}\right):=\;v_{i}(t_{ON})$$

#### NMDA spikes

As stated previously, the NMDAR system may be separated into slow (closing/current flow) and fast (opening/Mg^2+^ gating) subcomponents. Because the fast subsystem rapidly converges to the steady state, the gate's nullcline is stable while the channel is open with nullcline:

(7) $$B\left(V\right)=\frac{1}{1+e^{-\left(\frac{V-V_{s}}{k_{s}}\right)}}$$

Due to the strong time separation, we follow the tradition of considering the gating function to be instantaneous, hence defined by the nullcline (Jahr and Stevens, 1990a,b). In contrast, the slow subsystem inherits the channel's long closing time scale and describes current flow for leak and NMDAR components. For simplicity we only consider the leak and NMDAR components:

(8) $$C\frac{\partial V}{\partial t}=-\frac{V}{R_{m}}+gB\left(V\right)\left(E-V\right)$$

Here *g* denotes the max NMDAR conductance of the dendritic segment while *E* denotes the NMDAR reversal potential. As before, all potentials are translated for a resting potential of zero. In considering only NMDAR and leak current, it is necessary to increase the gating component's slope (decrease *k*_*s*_), less this reduction lead to global stability while the channel is open. Global stability would compromise the voltage dependence of spike production as glutamate binding would always result in an NMDA spike. Depending on parametrization, Equation (9) may have up to three equilibria, enabling bistability. Equilibria correspond to solutions of the implicit equation:

(9) $$\begin{array}{l} V_{eq}(x)=\frac{gE}{g+R_{m}{}^{-1}B{\left(V_{eq}(x)\right)}^{-1}}\\ \;=\frac{\left(\frac{gE}{g+R_{m}{}^{-1}}\right)}{1+\text{Exp}\left[-\frac{\left(V_{eq}(x)-V_{mid}+k\;ln[gR_{m}+1]\;\right)}{k}\right]} \end{array}$$

In the case of three equilibria, solutions possess locally stable lower (resting potential) and upper (saturation) equilibria. The middle equilibrium in this case is unstable leading to the “all or none” bifurcation in spikes. The single equilibrium case, in contrast, produces global stability, usually near the NMDAR reversal potential. As such, the single equilibrium case is pathological in the absence of other modulating voltage gated cation channels (VGC's) as glutamate binding would almost always produce an NMDA spike. However, in biological conditions, NMDA spikes still approach the NMDAR reversal potential, so the locally stable equilibria are roughly preserved. As long as three equilibria are maintained in the reduction to only leak and NMDAR dynamics, the locally stable equilibria remain accurate. To produce three equilibria, we simply modify the slope of Mg^2+^ blockade to compensate for the nonlinearity lost in removing other VGC's. However, it is important to note that full high-dimensional models include other equilibria due to Na^+^-spikelet's and, in the apical dendrite, Ca^2+^ spikes (see Antic et al., 2010). Also, although the stable equilibria are unmodified in the bistable case, the point of bifurcation (unstable equilibrium) may be. Biological evidence suggests other VGC's mediate both the threshold and amplitude of NMDA spikes such as Na^+^ (VGSC's) and Ca^+^ channels (VGCC's) as revealed with application of Na^+^ blocker TTX and Ca^2+^ blocker cadmium (Schiller et al., 2000). While the reduced dynamics still produce the correct amplitude for NMDA spikes, they may not produce the correct threshold in the absence of VGSC's/VGCC's. Hence, it may be necessary to additionally modify the midpoint of Mg^2+^ blockade. In the results section we describe when modification is and is not necessary due to the non-uniform distribution of spike thresholds (Major et al., 2008).

We make further reductions through the bifurcation of solutions. In assessing temporal dynamics after the initial channel opening, we consider the long time course of NMDAR bursts and clustering which give rise to macroscopic currents rather than the brief individual open durations Both decay and spiking occur on far shorter time scales than bursts, so states just prior to closing are almost binary and represent the nonlinear component of peak EPSP. As with the opening of individual channels, the population burst duration is considered multi-exponentially distributed (Gibb and Colquhoun, 1991, 1992; Wyllie et al., 1998) producing the expected value (*V*_*NMDA*_):

(10) $$\begin{array}{l} V_{NMDA}\left(x\right)=\sum_{i=1}^{n}\frac{\omega_{i}}{a_{i}}\int e^{-\frac{t}{a_{i}}}V\left(x,t\right)dt\\ \;=\sum_{i=1}^{n}\omega_{i}\left(\frac{R_{m}C}{R_{m}C+a_{i}}\right)[\frac{g}{C}\int e^{-\frac{t}{a_{i}}}B\left(V(x,t)\right)\\ \;\left(E-V(x,t)\right)dt+V_{0}] \end{array}$$

with ω_*i*_ the amplitude of the exponential component with slope *a*_*i*_. In present form, however, both the local dynamics (Equation 8) and expected NMDA component (Equation 10) lack explicit solutions in terms of ordinary functions. Using the bifurcation, we approximate Equations (8) and (10) by making Mg^2+^ blockade constant, following channel opening. In a fully dynamic regime, this method would not be justified. However, because we are only interested in which equilibria solutions approach, rather than how they get there, this method has fair accuracy, provided the earlier condition that Equation (9) has three solutions. As the slope of Mg^2+^ blockade increases (as was done to ensure bistability), the bifurcation point approaches the midpoint of B(V) (Equation 8). At the same time B(V) approaches a step function. The result is that the Mg^2+^ blockade approaches invariance except for an increasingly small region about *V*_*mid*_. Provided a sufficiently small *k* to ensure bistability, the Mg^2+^ blockade may then be approximated as invariant for initial points at channel opening sufficiently far from *V*_*mid*_. Changing the dynamic *B*(V) to a constant function of potential at channel opening *B(V*_0_*)* produces the following solution to Equation (8):

(11) $$\begin{array}{l} V_{NMDA}\left(x,t\right)\sim V_{0}(x)+\left(\frac{gE}{g+R_{m}{}^{-1}B{\left(V_{0}(x)\right)}^{-1}}-V_{0}(x)\right)\\ \;\left(1-Exp\left[-\left(\frac{gB\left(V_{0}(x)\right)+R_{m}{}^{-1}}{C}\right)t\right]\right) \end{array}$$

Hence with Mg^2+^ blockade constant while the channels are open Equation (10) becomes linear, so all solutions exponentially approach an equilibrium determined by the Mg^2+^ blockade at channel opening. We stress that this approach is only valid in approximating the path toward an equilibrium for Equation (8), with bistability induced by increasing the Mg^2+^ blockade slope. Because the transfer function only considers peak EPSP, this approach is sufficient for the current purposes but is not a valid approximation for the time course of fully dynamic dendrites. As an exponential, this equation is readily combined with burst length distributions. For a given starting potential, the ending potential with an n-exponential burst length distribution is itself n-exponentially distributed following translation. The expected value used in computing peak EPSP is:

(12) $$\begin{array}{l} V_{NMDA}(x)\;\sim \;V_{0}(x)+\left(\frac{gE}{g+R_{m}{}^{-1}B{\left(V_{0}(x)\right)}^{-1}}-V_{0}(x)\right)\\ \;\left[1-\sum_{i=1}^{n}\frac{\omega_{i}}{1+a_{i}\left(\frac{gB\left(V_{0}(x)\right)+R_{m}{}^{-1}}{C}\right)}\right] \end{array}$$

While we only present the case for multi-exponential closing distributions, the expected value is relatively insensitive to the type of distribution chosen as spiking and decay occur on much shorter time scales than the fall of NMDAR-mediated currents. When the NMDAR burst/cluster durations are considered sufficiently long, Equations (11) and (12) simplify to a simple sigmoid as in the artificial transfer function's non-linear component in Equation (2):

(13.1) $$V_{NMDA}\left(x\right)\sim \frac{gE}{g+R_{m}{}^{-1}B{\left(V_{0}(x)\right)}^{-1}}$$

(13.2) $$=\left(\frac{gE}{g+R_{m}{}^{-1}}\right)\frac{1}{1+\text{Exp}\left[-\frac{\left(V_{0}(x)-V_{mid}+k\;ln[gR_{m}+1]\;\right)}{k}\right]}$$

The second Equation (13.2) results from substituting the Mg^2+^ blockade Equation (7) and is the same as the equilibria Equation (9) when the Mg^2+^ blockade is assumed invariant between initial depolarization and its limiting equilibrium (spike or rest). As discussed previously, the number of starting points (initial depolarizations) for which this assumption is justified increases with the slope of Equation (7) (inversely proportional to *k*_*s*_). Hence, as Mg^2+^ blockade becomes increasingly binary, Equation (12) becomes an increasingly accurate description of NMDAR bistability. When the burst/cluster lengths are further assumed sufficiently long to approach limiting states (spike or rest), Equation (12) reduces to Equations (13). This reduction is greatly desirable as Equations (13) do not require explicit knowledge of burst length distributions.

#### Dendritic integration

In generating a (time-independent) transfer function, we sacrifice some information concerning the interaction of nonlinear components (NMDAR currents) in separate dendritic segments. Regardless of the number of incoming spikes, for instance, the induced somatic voltage would not be expected to significantly exceed the NMDAR reversal potential. Due to the continuous distribution of NMDAR's along the path of propagation, surplus depolarization would leak back through NMDAR channels before ever reaching the soma. However, the time independence of a transfer function prohibits fully dynamical propagation. While no individual spike crosses the NMDAR reversal potential using the method described above, summation of multiple spikes may, necessitating a boundary function as in Equation (1) to mimic dendritic saturation. Although the function *G(V)* provides a soft boundary, all other components of the function are unchanged. Hence the final transfer function for a given dendrite is the bounded sum of linear (fast ionic currents) and nonlinear (NMDAR-mediated) components. The final dendritic transfer function, *T*_*Bio*_*(v)*, may then be described explicitly in terms of the input vector *v* and decay vector δ:

(14.1) $$\delta_{i}:=e^{\left(-\;\frac{x_{i\to soma}}{\lambda }\right)}$$

(14.2) $$T_{Bio}\left(v\right)=G\left(\sum \delta_{i}\left[v_{i}+V_{NMDA}(x_{i})\right]\right)$$

Here λ is the functional length constant as in Equation (3), *V*_*NMDA*_*(x*_*i*_*)* is the nonlinear component for each input location, described by Equations (12) and (13) and *G(V)* is the linear-boundary function described in Equation (1). If desired, Equation (14.2) may be easily modified to allow differential spatial decay of spikes and subthreshold EPSP's. It is important to note that the boundary function to should be set to approach saturation with a single spike from the most proximal synapse, preventing the linear-summation of subthreshold EPSP's from applying to NMDA spikes in Equation (14.2). EPSP's between branches are allowed to sum linearly (Polsky et al., 2004) so the global transfer function is then the sum of individual dendritic transfer functions. For a single synapse with single-pulse stimulation, the use of an artificial boundary function is unnecessary as Equation (13.2) may be easily modified to the relative spike amplitude.

(15.1) $$\begin{array}{l} V_{Rel.Spike}=\left(\frac{gE}{g+R_{m}{}^{-1}}-v(0)\right)\\ \;\frac{1}{1+\text{Exp}\left[-\frac{\left(V_{0}(x)-V_{mid}+k\;ln[gR_{m}+1]\;\right)}{k}\right]} \end{array}$$

(15.2) $$T_{Single}=v_{0}+V_{Rel.Spike}$$

Here *v(0)* is the initial local depolarization. For a single synapse the sum of fast ionic currents and the relative spike approximates the (bounded) maximal EPSP amplitude. However, the sum of spikes, each bounded near the NMDAR reversal potential, does not necessarily share that boundary so the artificial boundary function is necessary for all nontrivial applications to compensate for the sublinear summation of spikes (Polsky et al., 2004).

### Parameterization

Throughout, parametrizations were generally that of Behabadi and Mel (2014): *R*_m_ = 10 KΩcm^2^, *E*_Leak_ = −70 mV (translated to 0 mV), *E*_NMDA_ = 0 mV (translated to 70 mV), *g*_(*NMDA*)_ = 3.9 nS. However, we used the conventional C = 1 μF/cm^2^ as opposed to Behabadi and Mel's unusually large capacitance of twice that much. The mid potential for NMDAR's was *V*_*s*_ = −23.7 mV (translated to 46.3 mV, Jadi et al., 2012). However, we only used 1/5 the typical value of *k*_*s*_ (2.5 vs. 12.5 mV, Major et al., 2008; Jadi et al., 2012), in order to compensate for the nonlinearity lost in computing expectations. This value was selected based upon the slope of target data (see **Figure 4**). To replicate double pulse data, the method of González et al. (2011) was utilized without modification. Durations prior to channel opening and burst/cluster lengths were based upon the multi-exponentials reported by Wyllie et al. (1998) for recombinant NR1a/NR2A after removing opening components less than 0.5 ms and burst/cluster length components less than 2 ms and reweighting. The filter on inter-opening times was to allow time for nonlocal inputs to reach the spike generation sites, while burst length distributions were filtered to place emphasis on the slower components which carry the vast majority of charge (Wyllie et al., 1998). NR1a/NR2A subunit was chosen due to its dominant role in spike generation (Polsky et al., 2009). Compartments for each input site had lengths of 10 μm and diameter 1 μm. To generate the observed small spike generation zone, the functional length constant for inter-compartmental contributions to spike generation was always half that of transients reaching the soma. Without this modification, the increasing threshold for spike generation with inter-electrode spacing would have to be on the same order as typical spatial decay which does not agree with the far greater effects observed empirically (see **Figure 4**).

### Simulations

To test the biophysical transfer function, we performed two sets of simulations custom-coded in MatLab2015a (Mathworks Inc., Natick, MA). In the first set of simulations we used a 5-state kinetic model of NMDAR's (Destexhe et al., 1998) to test appropriateness of the earlier separation assumptions. This choice was based upon the 5-state model's high temporal accuracy in describing NMDAR conformation changes which serves as a suitable contrast to a temporally agnostic transfer function. Membrane potential was modeled similarly to Equation (8) with NMDAR current multiplied by the probability of the channel having an open conformation. Membrane potential parameterizations during the kinetic simulations were identical to those used in the transfer functions, except for NMDAR maximal conductance which was set to the original 0.6 nS for the kinetic model (Destexhe et al., 1998) and inversely weighted for peak opening (~1/3) in the transfer function. Because input was concentrated on a single site, we used the full inter-opening distribution described by Wyllie et al. (1998). The initial Glutamate concentration was 1 mMol in the synaptic cleft for a single input instance with stimulation length 10 ms during which membrane potential was held constant. We compared peak membrane potentials to those predicted by Equations (12) and (13). The upper bound was set equal to the transfer function's “spike” peak amplitude.

In the second set of simulations, we simulated conditions of the seminal paper by Polsky et al. (2004) which was among the first to examine the location dependence of NMDA spike generation. Using whole-cell patch clamp recordings, the authors focally stimulated basal dendrites of cortical pyramidal cells using a pair of electrodes with spacings ranging from 20 to 200 μm. EPSP's from separate dendritic branches seemed to sum linearly at the somatic recording site, while separate EPSP's generated within a dendritic branch produced threshold-nonlinear interactions (NMDA spikes). The relevant analyses focused on the relation between stimulating sites within a branch individually and simultaneously. The “arithmetic sum” or “expected” peak EPSP was defined as the sum of individually evoked EPSP peaks and was compared to the “actual” peak EPSP elicited with simultaneous stimuli (**Figure 4**). Stimulation of dendrites can occur both in isolation (single pulse) or in combination, such as paired-pulse stimulations which produce more robust EPSPs than responses to single pulses. When varying inter-electrode spacing, we largely relied on paired-pulse stimulation (20 ms ISI), while a separate analyses was performed to compare paired and single-pulse stimulation with a fixed inter-electrode spacing (30 μm; **Figure 5**). All code is available online or by emailing the corresponding author.

## Results

### Kinetic model

**Figure 3:**
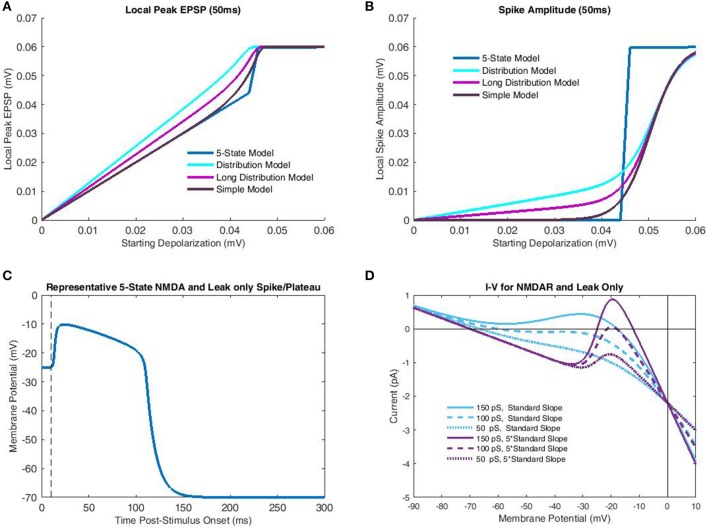
**Comparison of transfer functions with a 5-state kinetic model of NMDAR activation**. In the kinetic-model simulations parameterization of the membrane potential was identical to that of the transfer functions, which included the increased slope of Mg^2+^ blockade necessary for bistability. Like the transfer function only leak and NMDAR currents were considered. **(A)** Peak local EPSP's predicted for each model (single-pulse) are plotted as a function of depolarization at time of glutamate release. The 5-state model, simulated over a 50 ms interval for each case, largely demonstrates binary behavior, with a linear subthreshold portion, and a constant (spike) peak EPSP post-threshold. The “Distribution Model” corresponds to the transfer function with empirical cluster length distributions, while the “Long Distribution Model” uses a 50 ms mono-exponential distribution. The “Simple Model” only considers limit states and so is equivalent to an infinite burst/cluster length. The greater semblance of “Long Distribution” and “Simple” models to the 5-state model is due to the decreased dependence on fast-components which do not allow sufficient time for temporal decay to dominate nonlinear components. **(B)** Spike amplitude is plotted against depolarization at the end of Glutamate release for the same models as **(A)**. In all cases, the “Simple Model” of limit states bears greatest semblance to the 5-state model, particularly subthreshold, in which shorter burst-length distributions do not allow adequate temporal decay. **(C)** A representative NMDA spike/plateau time course simulated by the 5-state model with −20 mV membrane potential just following Glutamate release. Note that despite the proximity to the spike generation threshold in **(B)**, the 5-state model still predicts a rapid approach to spiking behavior. **(D)** Net current is plotted as a function of membrane potential for various combinations of NMDAR conductance and Mg^2+^ blockade slope. To achieve bistability (crossing 0 pA three times) it is necessary to have sufficiently large NMDAR conductance, and Mg^2+^ slope. With increased slope, modest levels of macroscopic conductance permit bistability, while for the standard slope, bistability is not attained for any conductance value.

Simulations with the 5-state kinetic NMDAR model generally supported the appropriateness of transfer function assumptions, provided a sufficiently large slope for Mg^2+^ blockade. As stated previously, NMDAR bistability relies on additional currents such as inward-rectifying K^+^ (Shoemaker, 2011) in biological settings. Using the standard slope for Mg^2+^ blockade, a system composed solely of leak and NMDAR currents will possess a single equilibrium (Figure 3D) corresponding to a spike save in cases of extremely low NMDAR conductance (in the current case < 150 pS; Figure 3D). As such, 5-state simulations used the same parametrization as the transfer functions (five times standard Mg^2+^ blockade slope). Simulations over a 50 ms period produced maximum peak EPSP's with the empirically observed “linear-hook” form described previously (Figure 3A). Three different transfer functions were simulated with all parameterizations identical except end time. The “Distribution Model” used the modified version of Wyllie et al. (1998) super-cluster lengths described previously. The “Long Distribution Model” used a single exponentially distributed (50 ms) burst/cluster length, while the “Simple Model” was as described in Equation (13) and only considers the limiting states (infinite burst/cluster duration). As should be expected, short burst/cluster lengths consistently over-predicted peak EPSP's as the local nonlinear currents did not have sufficient time to decay. The Simple Model, in contrast, only began to over-predict once approaching the Mg^2+^ midpoint near spike threshold. A simple and more accurate solution for the distributed closing time models would be use of a piecewise function making peak EPSP the maximum of linear (fast ionic) and nonlinear (NMDAR-mediated) components, rather than a bounded sum. Unfortunately, this approach is mathematically undesirable as it does not admit continuous derivatives of all orders. However, burst length distributions add little additional information due to the extremely short spike rise time (Figure 3C). As such, the Simple Model may also be more accurate in describing spike amplitude, particularly in subthreshold cases (Figure 3B). Overall, the rapid spike rise times (Figure 3C) and NMDAR bistability (Figure 3B) strongly support the time scale separations used in binary NMDAR open/shut states and bifurcations in Mg^2+^ blockade. In fact, results indicate that these factors may be exploited to an even greater extent, by further increasing the slope of Mg^2+^ blockade to approach the all-or-none spike threshold near Mg^2+^ blockade's midpoint (Figure 3B).

### Varying distance

**Figure 4:**
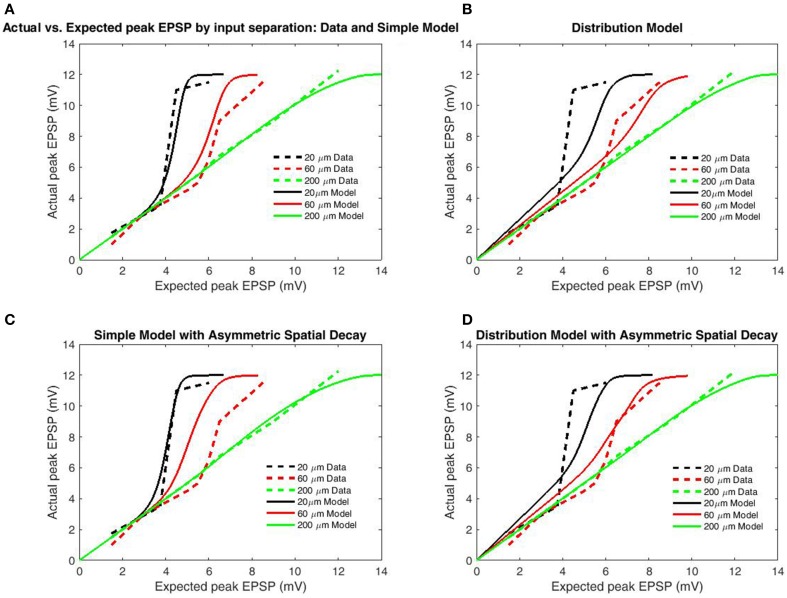
**Peak EPSP amplitudes for combined (“actual”) input are plotted against the sum of their independent contributions (“expected”) for varying distances between stimulation sites**. Data is redrawn from Figure 5C in Polsky et al. (2004). **(A)** Model of limit states (infinite burst length) with identical length constants for forward and back-propagation. **(B)** Model using empirical burst length distributions. **(C)** Model of limit states with increased length constant for backpropagation. **(D)** Model with burst length distributions with increased length constant for backpropagation. Direction-independent spatial decay appears to produce slightly better fits, while the limit-state model produces far better fits than the distribution-based model. The superior fit of the limit-based model is expected as the majority of charge in NMDAR bursting is carried by the slower components, while expectations based solely on the distribution over-weight the fast components.

To test the transfer function's accuracy, simulations were performed under the conditions of Polsky et al. (2004), described earlier. In all cases, we used a distance of 200 μm from the proximal input site to the soma, based upon the reported 80–250 μm range. While the proximal input site was fixed, distal input sites were varied to generate the 20, 60, and 200 μm inter-electrode spacings (Polsky et al., 2004). The boundary function was parameterized to match the observed boundaries (*b*_U_ = 12 mV, *b*_L_ = −12 mV, α_L_,_*U*_ = 0.5). The simulation design included two cases of model type (Simple Model and Distribution-based) and both symmetric and asymmetric spatial decay. A functional length constant of 77 μm has been reported for spikes/plateaus in basal dendrites propagating toward the soma (Major et al., 2008) and asymmetric length constants were tested using the spatial decay of back propagating action potentials (BAP; 138 μm; Nevian et al., 2007); (Figures 4C,D). Length constants for back-propagation were chosen based upon the BAP as the much larger length constants for unitary EPSP's could not allow the observed dependence on input spacing without additional spatial components (such as intracellular Ca^2+^ flow). As the distal ends of dendrites are “capped,” there is substantially less attenuation for potentials spreading distally. In both cases, the length constant for contributing to spike generation was half that of the respective functional length constant. Simulated results for the Simple Model (Figures 4A,C) well matched empirical data for both symmetric and asymmetric (separate backpropagation) length constants, indicating that a transfer function which only considers limiting states is sufficient to reproduce location dependence of peak EPSP's. However, the increased length constant for backpropagation decreased input spacing dependence, as should be expected as additional factors such as intracellular Ca^2+^ likely mediate the relationship. The model based on the distribution for burst/cluster lengths was slightly better with asymmetric length constants, but still mediocre in both cases (Figures 4B,D). As with comparison to the 5-state model (Figure 3), results demonstrate the addition of burst/cluster length is not only unnecessary, but detrimental.

### Paired vs. single-pulse

**Figure 5:**
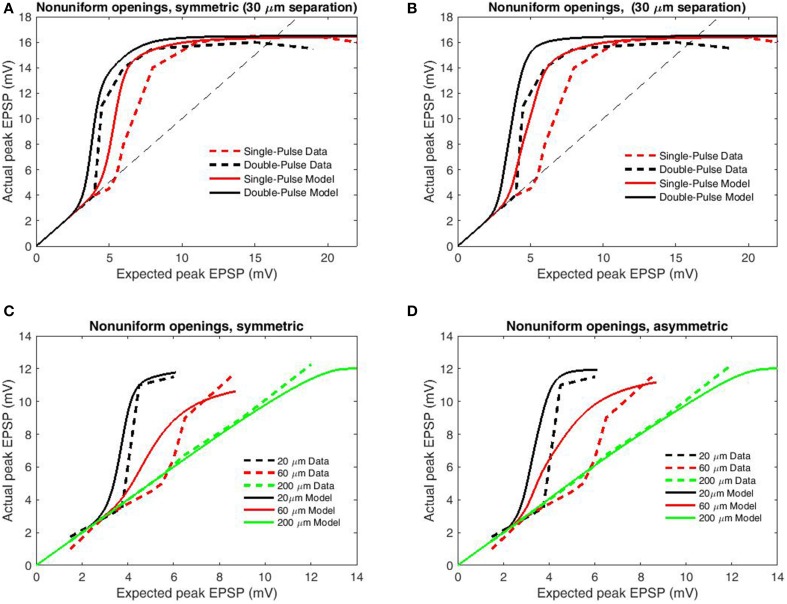
**Peak EPSP amplitudes for combined (“actual”) input are plotted against the sum of their independent contributions (“expected”) for paired-pulse and single-pulse stimulation methods at 30 μm inter-electrode distance**. Data are redrawn from Figure 4C in Polsky et al. (2004). **(A)** Model permitting fast (empirical) opening components upon the second pulse for primed synapses, symmetric spatial decay. **(B)** Modifications of **(A)** applied to the conditions of Figure 4A. **(C)** Same as **(A)** but with increased length constant for backpropagation. **(D)** Same as **(B)** but with increased length constant for backpropagation. Note that the inclusion of faster opening components for primed synapses and differential spatial decay for general and spiking transients well replicates the relation between paired-pulse and single-pulse stimulation without greatly compromising the relation with input separation for symmetric spatial decay. In contrast, these modifications are significantly deleterious when combined with asymmetric spatial decay, indicative that the spacing dependence of nonlinear dendritic integration displays less directional dependence than does voltage attenuation.

For a second analysis of transfer function accuracy, we compared simulated results for paired-pulse and single-pulse stimulation as by Polsky et al. (2004). Single-pulse protocols involve a single interval during which focal stimulation is delivered via an adjacent electrode, while paired-pulse protocols involve two stimulation intervals from the same electrode with very short ISI (in this case 20 ms). Paired-pulse stimulation is consistently superior in eliciting NMDAR-mediated currents, an effect known as paired-pulse facilitation (PPF) or NMDA priming. In accordance with the changed upper bound of data, boundary parameters were set as *b*_U_ = 16.5 mV, *b*_L_ = −16.5 mV. To further contrast PPF, synapses primed by the initial pulse are allowed, the previously removed fast components of the inter-opening distribution (the full distribution of Wyllie et al., 1998) which we term “non-uniform openings.” Based upon the previous results (Figure 4) only the simple model was used and the modifications employed in modeling PPF were applied to the previous simulations to determine generality. From these modifications paired-pulse stimulation results in decreased spike threshold relative to the individual components for both symmetric (Figure 5A) and asymmetric spatial decay (Figure 5B). While these additions successfully replicate the paired-pulse/single-pulse relationship, they do not greatly affect the transfer functions ability to account for input spacing with symmetric spatial decay (Figure 5C) and thus the simpler simulations on inter-electrode spacing remain valid (Figure 4). In contrast, the previous effects of asymmetric spatial decay are accentuated resulting in a poor fit of the relation with input spacing (Figure 5D). In summary, results demonstrate that the transfer function is accurate when either spatial decay or opening times are symmetric (or both). However, the contrast with single-pulse stimulation requires non-uniform opening times to mimic priming of NMDAR's by pre-bound glutamate After these adjustments the transfer function appears accurate for both paired and single-pulse stimulation for a wide range of input/spacing combinations.

## Discussion

**Figure 6:**
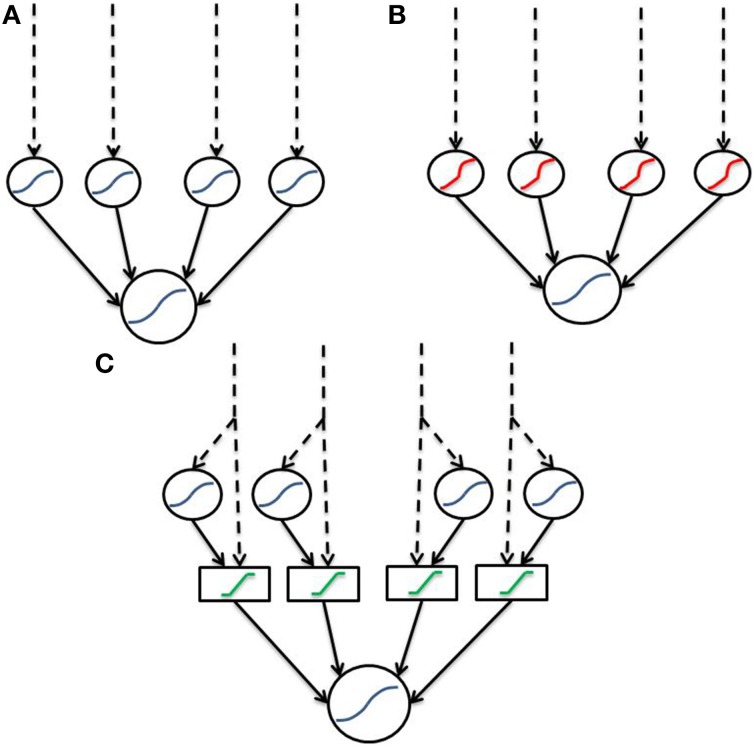
**Dendritic abstractions as neural networks**. Dashed lines indicate input, while solid lines indicate connections between layers. Blue functions are sigmoids, red are the previously described “linear hook” and green functions are boundary functions for saturation. **(A)** Standard two-layer model consisting solely of sigmoids. Contrary to data, the sigmoidal two-layer model does not include subthreshold linearity. **(B)** Two-layer with a “linear hook” type function which does contain subthreshold linearity. **(C)** “Two-and-a-half” layer decomposition of **(B)**. “Linear hook” type functions are decomposed into a linear (passive) and nonlinear (spiking) signal. These signals are then fed through a boundary function for dendritic saturation.

We have defined an artificial and a biophysical transfer function to model dendritic integration. Both functions are based upon sigmoidal opening dynamics of NMDA channels, however the biophysical function supports complex combinations of input, whereas the artificial function is agnostic to input location and simply considers a single nonlinear-component with each input equally weighted. Both transfer functions apply a bounded linear transform to the sum of linear and non-linear components to simulate saturation of the dendritic branch. Unlike many previous two-layer abstractions which describe sigmoidal components (Figure 6A) we implement a “linear hook” function to incorporate the observed subthreshold linearity (Figure 6B). In the current instantiation, the “linear hook” form results from two transformations akin to a “2-and-a-half layer” network (Figure 6C). In the first step, signals are split into a direct (passive) and indirect (active/spiking) pathway along the dendrite forming the “half layer.” In the second step, dendritic saturation allows signals within a band to pass unmodified, while those outside are greatly attenuated corresponding to the function *G(x)*. The result is a “linear hook” function as in Figure 6B with subthreshold linearity, extreme concavity post threshold and fairly hard boundaries.

Due to the spatial decay of post-synaptic signals within a branch, the distance between sites of stimulation is critical for determining the nonlinear threshold. As in Figure 4, increasing the site distance by 20–40 μm under the current parameterization drastically changes the current reaching the site of integration. However, the location dependence is reduced with high synaptic conductances (Cook and Johnston, 1999; Williams, 2005). Empirical results support this condition for apical dendrites of pyramidal cells (Williams, 2005). Hence, with high synaptic and low membrane conductances, the artificial transfer function increases in similarity to its biophysical counterpart. However, the biophysical function also uses the reversal potential of NMDA channels and hence contains its own dampening mechanism once local potentials pass this threshold. Due to the relatively high reversal potential of NMDA channels, the influence of this additional factor should be minimized when the evoked changes in potential are small, but near the dynamic range of the NMDA channel. The biophysical form also has a more complicated nonlinear component which includes products of differing sigmoidal function. When membrane capacitance is low or channel closing time is long, allowing the slow currents to quickly approach their equilibria, the biophysical nonlinear component again resembles the artificial sigmoid. As both the artificial and biophysical transfer functions saturate, they are trivially equivalent with extreme stimulation. From the view of computational complexity (hence processing time), the biophysical model requires significantly more computations than the artificial with many input locations within a branch. As each site is considered as a function of all other inputs as well as its own, the number of integration sites increases linearly with the number of inputs, and the number of computations per site similarly increases. Thus the biophysical transfer function offers the greatest advantage over its artificial counterpart when only a few inputs are considered. However, as stated before, both functions are computationally simple compared to time-dynamic models, so the difference in artificial and biophysical computation times is unsubstantial. It should be noted that that other reductions for dendrite-soma transfer exist for active dendrites with known conductance evolution (Wybo et al., 2013) as well as passive dendritic trees with specific geometries (e.g., van Pelt, 1992). In particular, the approach of Wybo et al. (2013) yields relatively low computation times compared with other models of dynamical dendrites with complexity characterized by a Fourier transform of a hyperbolic-trigonometric quotient. As with the current approach, use of transfer functions allow arbitrary dendritic morphology to be captured in the reduction of somatic voltage, in contrast to equivalent cable approaches (e.g., Ohme and Schierwagen, 1998). However, the current further reduction of approximating peak EPSP amplitude in terms of input, rather than the somatic-response kernel is orders of magnitude quicker (being explicit) which presents an alternative to point-node neurons without any increases in dimensionality or the presence of implicit relations which constrain mathematical analysis of the entire network (e.g. using methods of topological dynamics). As such, endowing a point-neuron with the current transfer function admits the same mathematical properties as the host somatic function, such as Poincaré-Bendixson properties for phase-plane analysis. Thus the current approach is particularly advantageous in adding dendritic morphologies to spiking neural networks which previously employed point-nodes. Problems in pattern recognition, for instance, admit a natural spatial hierarchy which may benefit from the addition of plausible dendritic morphology and input spacing to spiking nodes, without attempting full anatomical reconstruction.

Despite its simplicity, the biophysical transfer function is capable of replicating the sorts of non-linear interactions seen in pyramidal dendrites, which are typically expressed as systems of non-linear differential equations. Although some properties are lost in the use of a time-invariant function, such as capacitive membrane interactions, our replication of Polsky et al.'s findings (2004) greatly exceeds both the historical linear integration and the more recent model of sigmoidally integrative dendrites, particularly in the basal dendrites of pyramidal cells (Häusser and Mel, 2003; Polsky et al., 2004; Spruston and Kath, 2004). The focus upon fast components, naturally ignores dynamic changes in diffusion gradients, such as intracellular Ca^2+^ concentration, which lead to complex interactions in driving force between NMDA spikes and occur over variable time scales due to factors such as release from intracellular stores and pumping back into the extracellular fluid. However, based upon simulation results, the function appears well suited for its intended use in estimating peak somatic EPSP. Accurate modeling of dendritic integration takes on increasing importance as a growing body of evidence points to dendritic roles in areas of joint interest to biophysical and artificial neural network modelers, such as place fields and feature detection (Ujfalussy et al., 2009). Artificial networks may also benefit from replacing a layer of point neurons converging upon a single node with an artificial dendritic tree (Jadi et al., 2014) to greatly reduce dimensionality. As such, we hope this function will ease the computational demands of biologically-plausible dendritic integration while bridging gaps between artificial and biophysical models by allowing a smooth transition between forms.

### Conflict of interest statement

The authors declare that the research was conducted in the absence of any commercial or financial relationships that could be construed as a potential conflict of interest.

## References

[B1] Antic S. D. Zhou W. L. Moore A. R. Short S. M. Ikonomu K. D. (2010). The decade of the dendritic NMDA spike. J. Neurosci. Res. 88, 2991–3001. 10.1002/jnr.22444 20544831 PMC5643072

[B2] Behabadi B. F. Mel B. W. (2014). Mechanisms underlying subunit independence in pyramidal neuron dendrites. Proc. Natl. Acad. Sci. U.S.A. 111, 498–503. 10.1073/pnas.1217645111 24357611 PMC3890819

[B3] Branco T. Häusser M. (2010). The single dendritic branch as a fundamental functional unit in the nervous system. Curr. Opin. Neurobiol. 20, 494–502. 10.1016/j.conb.2010.07.009 20800473

[B4] Branco T. Häusser M. (2011). Synaptic integration gradients in single cortical pyramidal cell dendrites. Neuron 69, 885–892. 10.1016/j.neuron.2011.02.006 21382549 PMC6420135

[B5] Cook E. P. Johnston D. (1999). Voltage-dependent properties of dendrites that eliminate location-dependent variability of synaptic input. J. Neurophysiol. 81, 535–543. 10036257 10.1152/jn.1999.81.2.535

[B6] Destexhe A. Mainen Z. F. Sejnowski T. J. (1998). Kinetic models of synaptic transmission, in Methods in Neuronal Modeling, eds Koch C. Segev I. (Cambridge, MA: MIT Press), 1–25.

[B7] Genet S. Delord B. (2002). A biophysical model of nonlinear dynamics underlying plateau potentials and calcium spikes in Purkinje cell dendrites. J. Neurophysiol. 88, 2430–2444. 10.1152/jn.00839.2001 12424284

[B8] Gibb A. J. Colquhoun D. (1991). Glutamate activation of a single NMDA receptor-channel produces a cluster of channel openings. Proc. R. Soc. Lond. Ser. B Biol. Sci. 243, 39–45. 10.1098/rspb.1991.0007 1708142

[B9] Gibb A. J. Colquhoun D. (1992). Activation of N-methyl-D-aspartate receptors by L-glutamate in cells dissociated from adult rat hippocampus. J. Physiol. 456, 143–179. 10.1113/jphysiol.1992.sp019331 1293277 PMC1175676

[B10] Gómez González J. F. Mel B. W. Poirazi P. (2011). Distinguishing linear vs. non-linear integration in CA1 radial oblique dendrites: it's about time. Front. Comput. Neurosci. 5:44. 10.3389/fncom.2011.00044 22171217 PMC3214726

[B11] Häusser M. Mel B. (2003). Dendrites: bug or feature? Curr. Opin. Neurobiol. 13, 372–383. 10.1016/S0959-4388(03)00075-8 12850223

[B12] Jadi M. P. Behabadi B. F. Poleg-Polsky A. Schiller J. Mel B. W. (2014). An augmented two- layer model captures nonlinear analog spatial integration effects in pyramidal neuron dendrites. Proc. IEEE Inst. Electr. Electron. Eng. 102, 782–798. 10.1109/JPROC.2014.2312671 25554708 PMC4279447

[B13] Jadi M. P. Polsky A. Schiller J. Mel B. W. (2012). Location-dependent effects of inhibition on local spiking in pyramidal neuron dendrites. PLoS Comput. Biol. 8:e1002550. 10.1371/journal.pcbi.1002550 22719240 PMC3375251

[B14] Jahr C. E. Stevens C. P. (1990a). A quantitative description of NMDA receptor-channel kinetic behavior. J. Neurosci. 10, 1830–1837. 1693952 10.1523/JNEUROSCI.10-06-01830.1990 PMC6570302

[B15] Jahr C. E. Stevens C. P. (1990b). Voltage dependence of NMDA-activated macroscopic conductances predicted by single-channel kinetics. J. Neurosci. 10, 3178–3182. 1697902 10.1523/JNEUROSCI.10-09-03178.1990 PMC6570236

[B16] Major G. Polsky A. Denk W. Schiller J. Tank D. W. (2008). Spatiotemporally graded NMDA spike/plateau potentials in basal dendrites of neocortical pyramidal neurons. J. Neurophysiol. 99, 2584–2601. 10.1152/jn.00011.2008 18337370

[B17] Nevian T. Larkum M. E. Polsky A. Schiller J. (2007). Properties of basal dendrites of layer 5 pyramidal neurons: a direct patch-clamp recording study. Nat. Neurosci. 10, 206–214. 10.1038/nn1826 17206140

[B18] Ohme M. Schierwagen A. (1998). An equivalent cable model for neuronal trees with active membrane. Biol. Cybern. 78, 227–243. 10.1007/s004220050429 9602526

[B19] Poirazi P. Brannon T. Mel B. W. (2003). Pyramidal neuron as two-layer neural network. Neuron 37, 989–999. 10.1016/S0896-6273(03)00149-1 12670427

[B20] Polsky A. Mel B. Schiller J. (2009). Encoding and decoding bursts by NMDA spikes in basal dendrites of layer 5 pyramidal neurons. J. Neurosci. 29, 11891–11903. 10.1523/JNEUROSCI.5250-08.2009 19776275 PMC3850222

[B21] Polsky A. Mel B. W. Schiller J. (2004). Computational subunits in thin dendrites of pyramidal cells. Nat. Neurosci. 7, 621–627. 10.1038/nn1253 15156147

[B22] Rall W. (1969). Time constants and electrotonic length of membrane cylinders and neurons. Biophys. J. 9, 1483–1508. 10.1016/S0006-3495(69)86467-2 5352228 PMC1367649

[B23] Schiller J. Major G. Koester H. J. Schiller Y. (2000). NMDA spikes in basal dendrites of neocortical pyramidal neurons. Nature 404, 285–289. 10.1038/35005094 10749211

[B24] Shoemaker P. A. (2011). Neural bistability and amplification mediated by NMDA receptors: analysis of stationary equations. Neurocomputing 74, 3058–3071. 10.1016/j.neucom.2011.04.018

[B25] Spruston N. Kath W. L. (2004). Dendritic arithmetic. Nat. Neurosci. 7, 567–569. 10.1038/nn0604-567 15162161

[B26] Ujfalussy B. Kiss T. Érdi P. (2009). Parallel computational subunits in dentate granule cells generate multiple place fields. PLoS Comput. Biol. 5:e1000500. 10.1371/journal.pcbi.1000500 19750211 PMC2730574

[B27] van Elburg R. A. J. van Ooyen A. (2010). Impact of dendritic size and dendritic topology on burst firing in pyramidal cells. PLoS Comput. Biol. 6:e1000781. 10.1371/journal.pcbi.1000781 20485556 PMC2869305

[B28] van Pelt J. (1992). A simple vector implementation of the Laplace-transformed cable equations in passive dendritic trees. Biol. Cybern. 68, 15–21. 10.1007/BF00203133 1486128

[B29] Wainrib G. Thieullen M. Pakdaman K. (2012). Reduction of stochastic conductance-based neuron models with time-scales separation. J. Comput. Neurosci. 32, 327–346. 10.1007/s10827-011-0355-7 21842259

[B30] Williams S. R. (2005). Encoding and decoding of dendritic excitation during active states in pyramidal neurons. J. Neurosci. 25, 5894–5902. 10.1523/JNEUROSCI.0502-05.2005 15976078 PMC6724799

[B31] Wybo W. A. M. Stiefel K. M. Torben-Nielsen B. (2013). The Green's function formalism as a bridge between single- and multi-compartmental modeling. Biol. Cybern. 107, 685–694. 10.1007/s00422-013-0568-0 24037222

[B32] Wyllie D. J. A. Béhé P. Colquhoun D. (1998). Single-channel activations and concentration jumps: comparisons of recombinant NR1a/NR2A and NR1a/NR2D NMDA receptors. J. Physiol. 510, 1–18. 10.1111/j.1469-7793.1998.001bz.x 9625862 PMC2231013

